# Neuromodulation of the lingual nerve: a novel technique

**DOI:** 10.3171/2020.7.FOCVID2018

**Published:** 2020-10-01

**Authors:** Kevin Zhao, Christopher E. Talbot, Antonios Mammis, Boris Paskhover

**Affiliations:** 1 Departments of Neurological Surgery and; 2 Otolaryngology–Head and Neck Surgery, Rutgers University, New Jersey Medical School, Newark; Saint Barnabas Medical Center, RWJ Barnabas Health, Livingston, New Jersey

**Keywords:** lingual nerve neuropathy, lingual nerve neuromodulation, lingual nerve stimulation

## Abstract

The lingual nerve is a branch of the posterior trunk of the mandibular nerve. It provides sensation and taste to the ipsilateral anterior two-thirds of the tongue. Posttraumatic neuropathy of the lingual nerve can be chronic and debilitating long after the inciting trauma. In this operative video, the authors describe a novel technique for the treatment of lingual nerve neuropathy with neuromodulation. They present a case of a 69-year-old female with posttraumatic lingual nerve neuropathy after left molar extraction. The patient reported 95% symptom improvement after the procedure. This video demonstrates the feasibility of lingual nerve neuromodulation.

The video can be found here: https://youtu.be/l-CKP8-8eqk

## Transcript

This is a video demonstrating a new surgical technique for treatment of posttraumatic lingual nerve neuropathy. In this video, we describe a novel surgical technique for treatment of chronic posttraumatic lingual neuropathy.

**0:35 Lingual Nerve.** The lingual nerve is a branch of the posterior trunk of the mandibular nerve. It courses along the medial aspect of the ascending ramus of the mandible. It provides sensation and taste to the ipsilateral anterior two-thirds of the tongue. The lingual nerve comes in close proximity to the third molar, rendering it prone to injury during third molar extraction and other dental procedures. Posttraumatic neuropathy of the lingual nerve can be chronic and debilitating long after the inciting trauma. Patients may experience a wide range of symptoms, including pain, numbness, tingling, burning sensation, and dryness.

**1:17 History and Examination.** Patient is a 69-year-old female with a past medical history of a left molar extraction 3 years prior. She now presents to the functional neurosurgery clinic with a complaint of progressive and constant severe pain, numbness, tingling, dryness, sour taste, and a “pulling sensation” on the left half of her tongue. Her symptoms were exacerbated by chewing and speaking and were alleviated with sleep and rest. These symptoms had a deleterious effect on her activities of daily living, such as eating and brushing her teeth. Gross and neurological examinations of her face, tongue, and oral cavity with a focus on the function of cranial nerves V, VII, IX, X, and XII were unremarkable. She had attempted conservative management including medications without any relief. Conservative medical therapy options were exhausted, and no single therapy provided substantial relief of her symptoms.

**2:18 Diagnosis and Treatment.** More invasive therapies were attempted and included multiple targeted nerve blocks by the otolaryngology team along the ascending ramus of the left mandible. This provided significant but temporary relief of symptoms. The patient also underwent an endoscopic-assisted left lingual nerve decompression and partial ablation. This provided temporary symptom relief. This has served both as a treatment modality as well as diagnostic maneuver to localize her symptoms to the left lingual nerve.

**2:51 Lingual Nerve Neuromodulation.** After failure of multiple treatment modalities, discussion was held and surgeons proposed a neuromodulatory procedure to implant a stimulator electrode along the course of the left lingual nerve. The patient understood the procedure was novel and experimental, and she agreed to proceed. The procedure was planned as two stages. The first in which the electrode would be implanted and leads externalized to test the stimulation efficacy. The second stage would be implantation of a pectoral generator. Preoperative studies were performed in a cadaver lab to ensure anatomical feasibility. The potential risk of lead migration was addressed through submental introduction of the lead and anchoring to the mandibular periosteum. This allowed the lead and mandible to move as a single unit and reduce the risk of lead migration with mastication, speaking, and other mandibular movements (Supplemental Figs. 1 and 2).

**3:48 Surgery.** The patient was placed supine on a regular table with her arms tucked. Submental and pectoral incisions were marked for lead introduction and externalization, respectively. Under direct endoscopic visualization, a 2-cm incision was made along the left mandibular ramus using monopolar electrocautery. Subperiosteal elevation was performed along the medial segment of the jaw. Dissection was performed to identify the medial pterygoid muscle and epineurial fascia underlying the vertical segment of the lingual nerve. Fascia and scar tissues were carefully released longitudinally and circumferentially along the course of the nerve. With the nerve fully decompressed and exposed, the submental incision was opened and dissected down to the bone. A disposable lead passer was introduced through the submental incision and carefully advanced into the intraoral exposure, while staying within a subperiosteal plane and respecting the lingual nerve. A linear array stimulator was then passed through the sheath and positioned alongside the lingual nerve. An anchoring device was used to anchor the electrode to the mandibular periosteum. The lead passer and sheath were then used to pass the distal portion of the stimulator lead subcutaneously to the pectoral incision. This was then connected to an extension wire, which exited at the skin and was connected to the external generator for further testing.

**5:58 Intraoperative Monitoring and Postoperative X-Ray.** Intraoperative neuromonitoring demonstrated firing of the temporalis muscle while the stimulator was turned on, indicating adequate proximal positioning of the lead to the muscular branches of V3. Lead placement was further confirmed with postoperative x-ray (Supplemental Figs. 3 and 4).

**6:17 Outcome.** With activation of the generator in the postanesthesia unit, the patient reported immediate symptom improvement of 95%. At home, she reported substantial relief while brushing teeth and eating. The patient underwent placement of a permanent pectoral generator 1 week after the initial lead placement. At 3 months’ follow-up, the patient had no complications, reported persistent and stable symptom relief.

This video demonstrates the feasibility of lingual nerve neuromodulation and a novel technique to reduce the risk of lead migration. Longer follow-up will be needed to study the sustainability of symptom relief as well as long-term complications.

**6:54 References** ^1–6^

## Author Contributions

Primary surgeon: Paskhover, Mammis. Assistant surgeon: Zhao, Talbot. Editing and drafting the video and abstract: Zhao, Talbot. Critically revising the work: all authors. Reviewed submitted version of the work: all authors. Approved the final version of the work on behalf of all authors: Paskhover. Supervision: Mammis.

## Supplemental Information

### Online-Only Content

Supplemental material is available online.

*Supplemental Figs. 1–4*. https://thejns.org/doi/suppl/10.3171/2020.7.FOCVID2018.

## Supplemental Information

Supplemental Figures
