# Supplementary material for: Neuromodulation of the lingual nerve: a novel technique
Source: Neurosurg Focus Video. 2020 Oct 1;3(2):V6. doi: 10.3171/2020.7.FOCVID2018 (PMC9542482; doi:10.3171/2020.7.FOCVID2018)
Supplement: Supplemental Figures [file SupplementalFigs1-4_FOCVID20-18.pdf]

NEUROSURGICAL FOCUS VIDEO

ONLINE ONLY

Supplemental material

Neuromodulation of the lingual nerve: a novel technique

Zhao et al.

https://thejns.org/doi/abs/10.3171/2020.7.FOCVID2018

DISCLAIMER The *Journal of Neurosurgery* acknowledges that the following section is published verbatim as submitted by the authors and did not go through either the *Journal's* peer-review or editing process.

© 2020 The authors, CC BY 4.0 (http://creativecommons.org/licenses/by/4.0/)

Supplemental Figure 1:

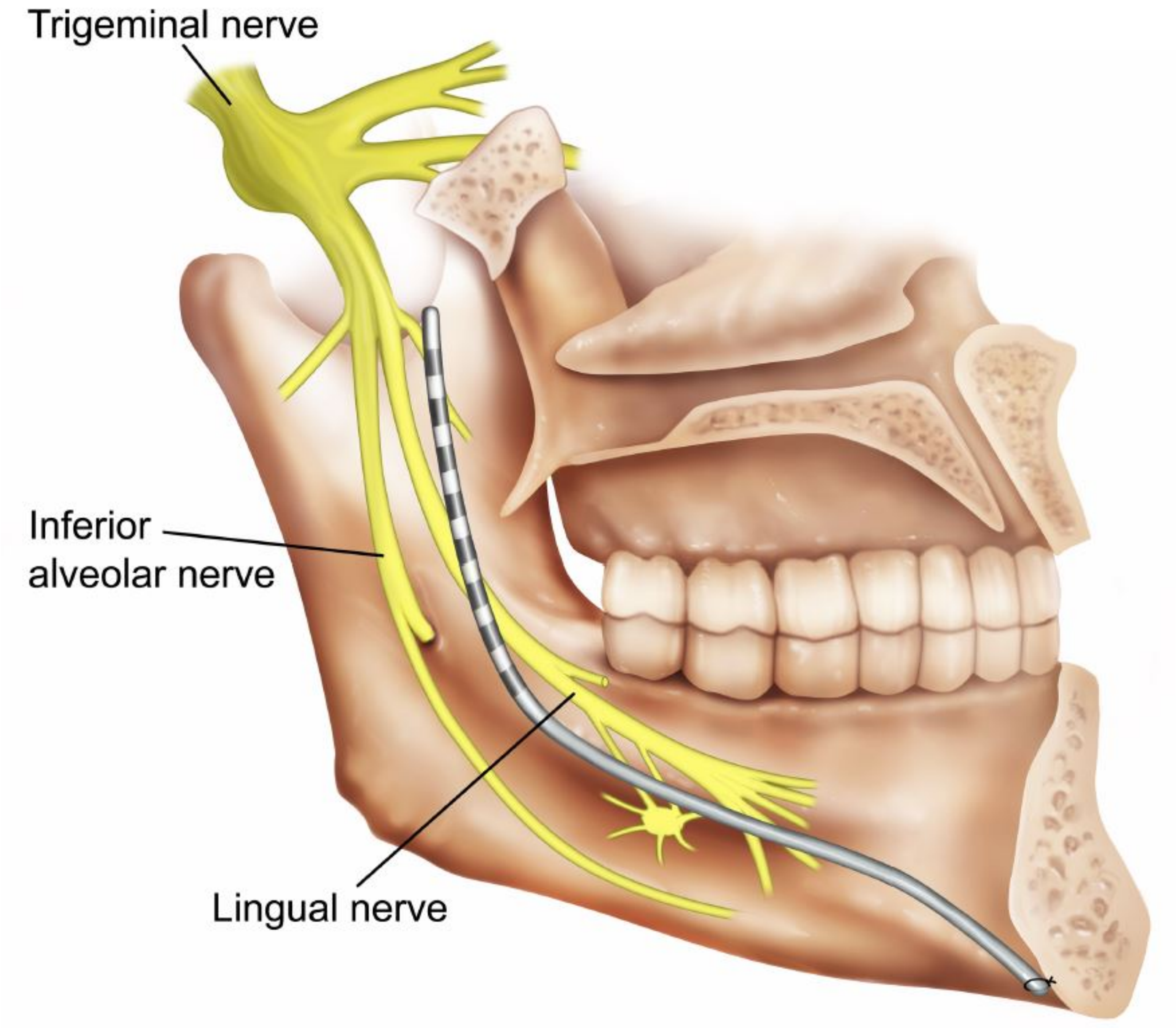

Supplemental Figure 1: Illustration demonstrating the placement of the electrode in relation to the lingual nerve along the ascending ramus.

Supplemental Figure 2:

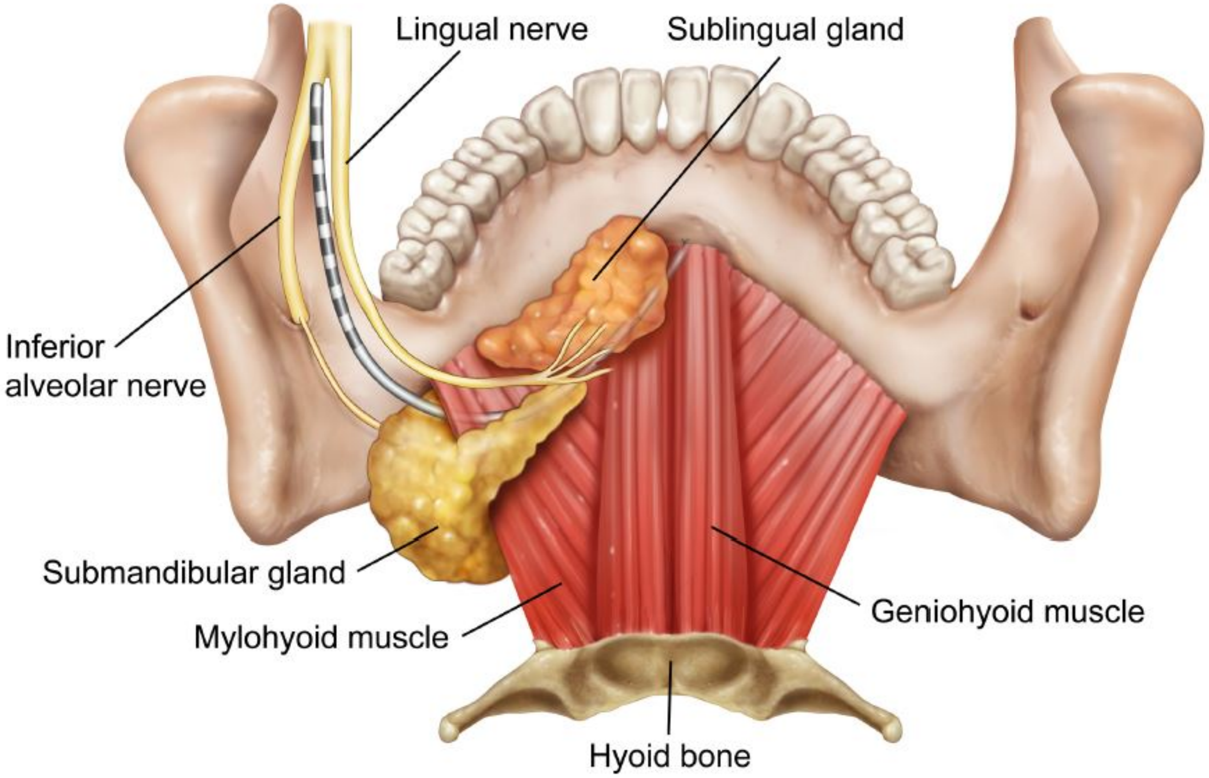

Supplemental Figure 2: Illustration demonstrating the placement of the electrode in relation to the lingual nerve along the ascending ramus.

Supplemental Figure 3:

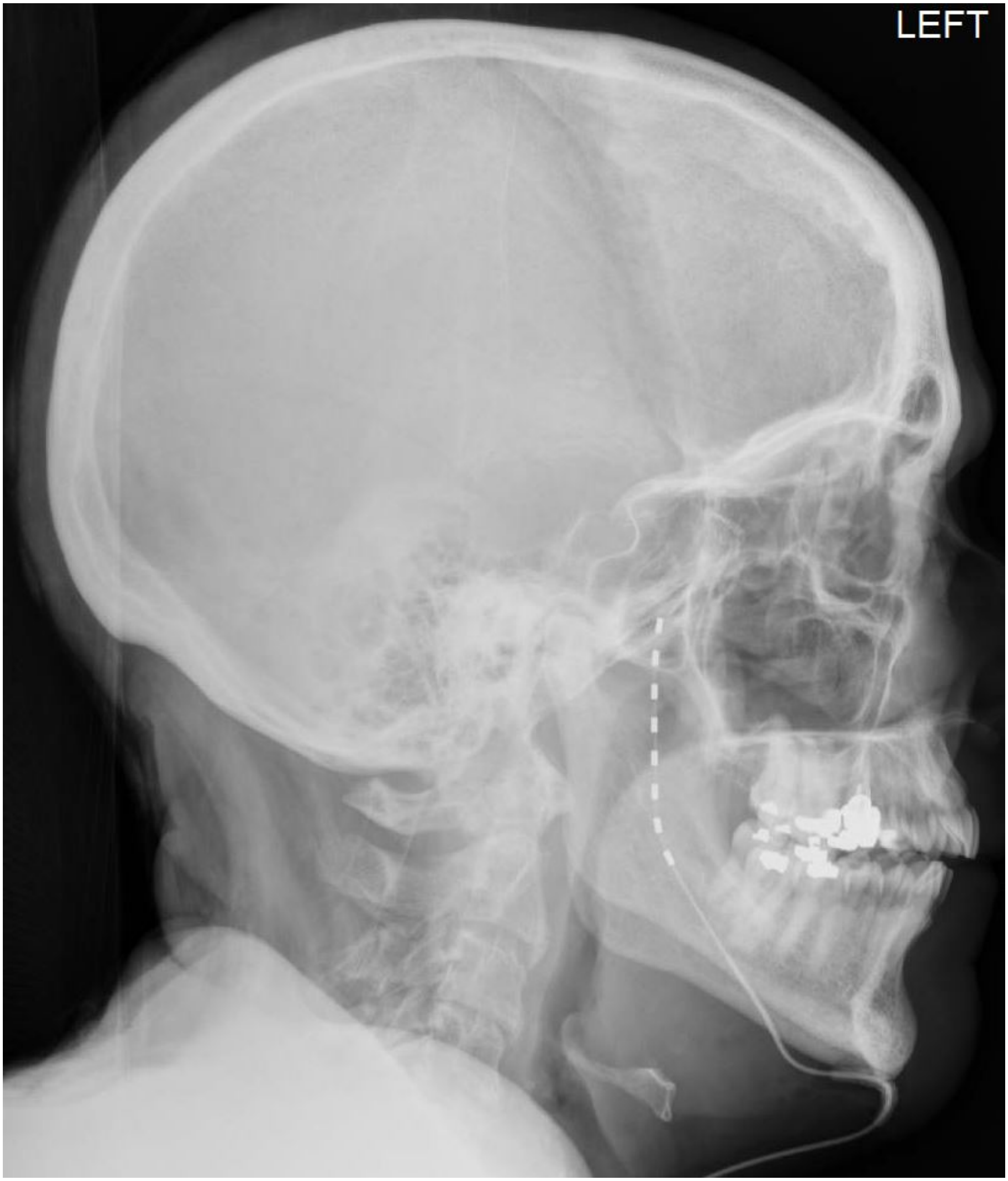

Supplemental Figure 3: AP plain film demonstrating the correct position of the electrode along the left ascending ramus.

Supplemental Figure 4:

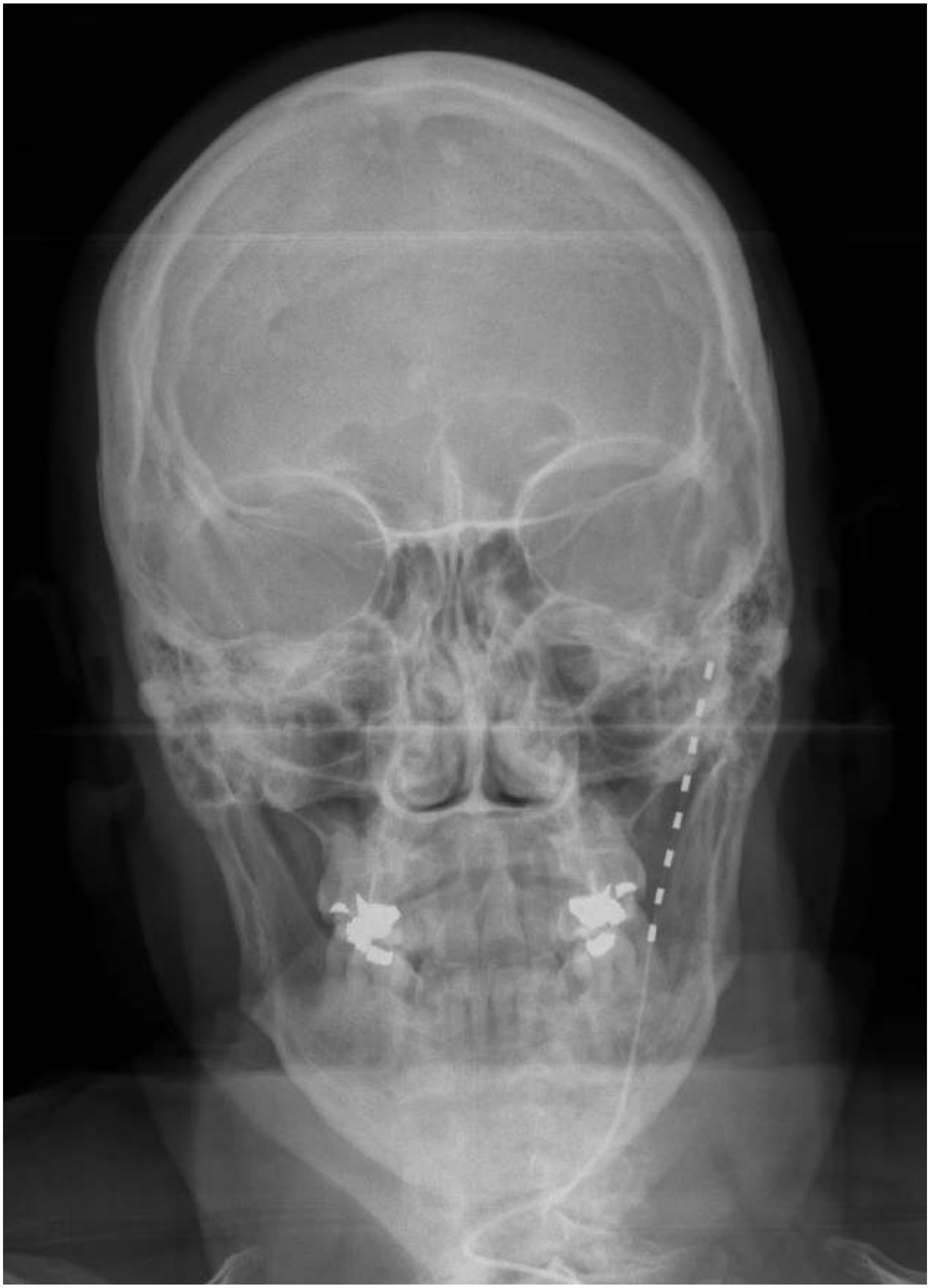

Supplemental Figure 4: Lateral plain film demonstrating the correct position of the electrode along the left ascending ramus.
